# Medical Societies in the Country

**Published:** 1879-01

**Authors:** E. A. Cobleigh

**Affiliations:** Athens, Tenn.


ATALNTA
Medical and Surgical Journal.

VOL. XVI.] JANUARY—1879. [NO. 10

### Original Communications.

### MEDICAL SOCIETIES IN THE COUNTRY.

BY E. A. COBLEIGH, M.D., ATHENS, TENN.

I beg the indulgence of editor and reader that I may say a few words on that much worn topic of medical societies. It is not my purpose to attack defects, or suggest reforms, for those already existing. I say God speed them in the work they are doing. They have accomplished much in the past, and a vast field of usefulness for them presents in the future. Nor do I propose suggesting anything whatever to my medical brethren who are active members of live professional organizations. It is a plea for the forming of new societies, and especially for the pushing of this work by and among the country practitioners, that I enter here.

Not a large center of population in our country but has from one to a score of medical societies in its midst. This is as it should be, and many new points of practical importance are given to us, as a profession, first in the transactions of these very bodies. But ought the good work to stop here? Emphatically no. In these medical centres—the large cities of the United States—hospitals, colleges, museums, lectures on specialties, and various other opportunities for self-information, present themselves for the benefit of him who is seeking greater excellence in medical science. Therefore the need for societies seems to be trifling. Yet they spring up, as it were, spontaneously, to fill some place not occupied by these other sources of knowledge, which is felt and appreciated by the profession. And, as a rule, when once born, they grow, flourish and do good.

Now, while this state of things obtains in our cities, and in *some* of our towns and villages, there are hundreds of points where no societies are to be found. This is particularly true of East Tennessee, and our section here is only one of many in the same condition. Why does not a change occur? I can see no valid reason. To the sloth and self-blindness of my own profession belongs the blame, and I do not wonder that the standard of medical perfection fails to rise higher among country practitioners while they persist in slighting chances for improvement which are within such easy reach.

If there is any professional man who, above others, ought to be a paragon of perfection in wisdom, patience, skill, politeness and other virtues too numerous to mention, it is the physician. In his hand he holds, to a certain extent, the lives, health and happiness of this great humanity around us. How much, then, hangs on his shoulders! He who lightly assumes and carries this load of responsibility, is unworthy of the title he bears.

Nor is there to-day any branch of science which is striding more rapidly onward than medicine. Each day seems to bring new discoveries or some improvement in our means of diagnosis, or therapeutics. He who would keep pace with these rapid changes, must avail himself of *every* opportunity he can obtain to post himself thereon, and unless he does this, both his wisdom and his success soon become things of a past age, an obsolete era of the world's progress, unsuitable for comparison with the living present.

Now, there is no class of practitioners more favorably situated for falling behind in this great march of science than those who are located in our villages and cross-road settlements. Almost, or quite, cut off from the advantages enjoyed by their city brethren, either never seeing other medical men or meeting them so seldom that few opportunities for exchange of opinions occur, making long, lonely trips through sparsely settled regions, given little time for reading, in many cases debarred by impecuniosity from reaping the benefits of a good library, they only too frequently lead isolated lives professionally—cut off, as it were, from the entire medical world. Some, it is true, are enterprising men, and, as best they can, they study their science and art. They buy new books, take and read the leading journals, put what is new and practica into use, and make intelligent, capable doctors. So far, so good. Is there nothing better? Why not bring the ignorance of one of these classes into contact with the education and culture of the other and elevate the former, also, This can be done, it has been, and will be again.

Now, what are the influences which work to prevent association among neighboring physicians, and their assembling together for social and professional confab at frequent periods? Sometimes, lack of acquaintance with each other. Sometimes, business rivalry, involving jealousy of reputation. Often the plea is press of business. And, lastly, the lack of regular organization into societies. Let us dissect these reasons for not enjoying the pleasures and reaping the benefits of society membership and attendance. The first excuse is a poor one, for it is no difficult matter to make acquaintances when we really wish to. If you or I, kind reader, wanted to form a society, we would find it very little trouble to call on a person, even if a stranger, whom we desired to co-operate with us in the movement, and to state the object of our visit to him. In nine cases out of ten we would be politely received and glad that we went. And, if we felt uninclined to go in person, a written message would answer nearly, or quite, as well. So we find excuse number one to be just no excuse at all.

Second, jealousy stands often in the way of rival physicians affiliating together. Each has, or thinks he has, one or more grievances against his competitor. This is an unpleasant state of affairs to both. Often the apparent grounds of dislike are not founded on fact at all. If left alone these ruptures between competitors usually widen by degrees, each one slurring the other when occasion presents, until at length the breach becomes perpetual—hopeless of cure by any human intervention. Thus two lives are embittered, frequently from no *real* cause of offense at first, but from baseless rumors of injury or insult which "so and so says" has occurred. More frequently a careless remark, unintended to do harm, reaches a rival's ear, is misinterpreted, a retaliating spirit spurs tongue and actions to resent the supposed slur, and coldness deepens to frigidity between the two. Now, if some energetic doctor would inaugurate a medical society, and get these enemies into it, the chances are that a mutual apologizing would occur sooner or later, misunderstandings be cleared up, and a better state of feeling supervene. Nothing so speedily or effectually breaks down these professional animosities as do our society meetings. I have seen this result occur repeatedly during my connection with the several medical organizations which fortune has at different times enabled me to have membership with. In a few cases I have even known brother physicians who would purposely pass on the streets with studied indifference, become, after a few months, the warmest of friends, in this very way; and that, too, when neither, at the beginning, would deign to take part in any discussion into which the other had already entered. Never but once have I seen society membership fail to work a greater or less improvement in the mutual relations of antagonistic practitioners. In that case the rival surgeons had their names on the roll of an association, meeting once a week. Without any arrangement between them to that effect, it always occurred that one came to the sessions of the body one week and the other the next, and thus they alternated for several years, continuing the habit up to the present time. Thus we see that, as a rule, excuse number two, like number one, proves no objection to organization, but rather tends to render it desirable.

Third, press of business. God speed the overworked physician in his mission of philanthropic healing. I would not add a straw to his already heavy burden. But is it a burden to keep up membership with a live society ? Is it not a duty that ought to be of a pleasant nature ? My brother-laborer, if you are doing so much business do you not owe it to your patrons, to yourself, and to the great cause whose devotee you are, to give and receive all the wisdom you can for the better performance of those very duties you daily engage in. If so busy, then probably you have little time for reading. Make it up by devoting at least one day per month to society attendance. If you are already old you cannot fail to learn *something* new, which may enable you to simplify or improve your methods of labor so as to gain time, lighten your work, or at least do your patients full justice by giving them the benefits of all that is known, or done, in cases similar to theirs. He who knows enough has lived out his day of usefulness, and is in a complacent state of mind most favorable for immediate translation to another sphere. He ought to go at once, ere he again grows ambitious, and covetous of something more. Again, if you have grown gray as a busy practitioner you have enjoyed a wide field of observation and experiment, and you possess a valuable fund of information. As you, at first, freely received from your predecessors the knowledge on which your career began, so you owe it to your younger brethren to meet them, and impart to them such new and useful ideas as you can. So "too busy" is no good reason for holding aloof from medical societies. The busier one is, the greater his obligation to learn and to teach. If he cannot attend monthly society meetings let him go every two months or every quarter as he can. He will, in the end, find himself well repaid for the trouble and time thus spent.

Lastly, we come to consider the oft-urged plea of no organization. "We have no society in our leculity," is the ostensible reason for not reaping the benefits of one. Why have you none? Dr. A., or Dr. B., or Dr. C. have never tried to form any, or have failed in the effort. Well, is it any more Dr. A., B. or C's. business to take the initiatory than your own? Not at all; particularly not; if you really want one in your locality. *Go at it yourself.* If you are for sometime established where you are, write out a call for a preliminary meeting, at a certain time and place, to consider the feasibility of organization, and head the list of callers with your own name. Then get two or three other practitioners to sign said call with you. If you can not see them in person, get their assent by mail, and issue your call in the nearest newspaper. Lacking a paper, use the mail again, or the village post-office door, or any public place. Publish it. Get word to every physician within a radius of twenty-five or thirty miles, and I will guarantee that at the appointed time a sufficient number of doctors will put in an appearance to constitute a respectable nucleus for future growth.

If you are a junior practitioner in the locality do not be deterred from action when the seniors fail to move. Write out your call, get the older physicians to sign it, then put your own name at the foot of the list and publish it. This last cause is the most prolific preventive of the organization of societies —each one waits for another to move. When I returned to my present location from a Western residence, I felt badly to give up my active connection with the medical society there, and come to a place where none existed. I determined not to be debarred from society privileges. Coming to a section where no association of the kind had ever existed, I early wrote my call, got four physicians out of six in my town to sign it, got consent to use the names of several others at distant points in the county and issued the document. Eight or ten doctors came to our meeting and the Hiwassee Medical Society had its birth then and there with no flattering prospects, and but a handful of members. Month by month it grew, by a little exertion on our part, and, after only eighteen months of existence, it to-day embraces in its territory five counties and a goodly membership. Thus was accomplished here, where professional strife ran unusually high, what the medical fraternity had individually longed for for twenty years or more, but what had never been taken hold of in earnest by any one. It was a matter of no trouble at all to light the spark, which early grew to a flame. Any one could have done it as well, some better, than myself. Each month we come together and enjoy a day of social pleasantry and professional refreshing. Old feuds have been forgotton, grievances forgiven, and we are welded into a common brotherhood for mutual improvement and the sustaining of our common dignity as medical men.

One month we meet at one point, the next at another, so that our most distant members may enjoy occasional meetings near home. Sometimes we have to ride fifteen, twenty, or more, miles, by rail or overland, in heat and cold, wet and dry, but we go just as often as we can, and feel amply repaid for the inconvenience we undergo to be present. And this can be done anywhere. In town and country the physicians are ready to organize if but one energetic mind will take hold of the matter and set the ball in motion. No one can imagine the revolution in professional affairs which such a movement occasions, especially in country sections where none has taken place before. To witness it is the only way to be convinced.

Then I would urge upon my fellow physicians in such localities to try it. Take action personally, vigorously and with patience where much effort is needed, persist in agitating, get your societies formed and then attend them regularly if possible. Let nothing but the most imperative demands cause your absence. Take part willingly in the proceedings, no matter in how humble a manner. Teach your brethren what you can. If you have nothing to teach them, then learn from them, but do both when it is possible. If your membership chances to be too scattered for monthly meeetings to be practicable, meet every two, three or four months as circumstances will warrant. But have your sessions with regularity, and as a rule, the more frequent they are, the more interest will be manifested, and the better will your attendance be.

Try it, at least. If failure come no harm results. When one organization is effected others usually follow soon in the neighborhood. The profession becomes a unit with single purposes; quackery and divers impositions on the public credulity can be authoritatively and with unison decried, exposed or stamped out; you who were before bitter rivals, will be astonished to find how pleasant association with your peers is; backbiting ceases, ethics obtain the ascendancy, fees can be regulated, the general public enlightened on sanitary subjects, and thus your duties made easier and pleasanter, and regular practitioners who fall into irregular habits can be frowned down and driven into private life. And, as societies multiply, many and much needed reforms in medical polity, medical education, etc., can be more readily brought about, by co-operation among these bodies and the welding together of the great body professional, at present so scattered, yet having common interests all over the world can be had. Let us move, then, and move with a will. Let the country come up to the help of our cities, let the towns and villages, the hills and valleys, the prairie and the woodlands muster their little corps for self-improvement and the up-building of our glorious science by concerted and harmonious action.

